# Fulvestrant treatment of precocious puberty in girls with McCune-Albright syndrome

**DOI:** 10.1186/1687-9856-2012-26

**Published:** 2012-09-22

**Authors:** Emily K Sims, Sally Garnett, Franco Guzman, Françoise Paris, Charles Sultan, Erica A Eugster

**Affiliations:** 1 Section of Pediatric Endocrinology/Diabetology, Riley Hospital for Children, Indiana University School of Medicine, 705 Riley Hospital Drive, Room 5960, Indianapolis, IN 46202, USA; 2 AstraZeneca, Macclesfield, United Kingdom; 3 Former-AstraZeneca, Wilmington, Delaware, USA; 4 Pediatric Gynecology and Endocrinology, University Hospital of Montpellier, Montpellier, France

**Keywords:** McCune Albright syndrome, Peripheral precocious puberty, Estrogen receptor antagonist

## Abstract

**Background:**

McCune-Albright Syndrome (MAS) is usually characterized by the triad of precocious puberty (PP), fibrous dysplasia, and café au lait spots. Previous treatments investigated for PP have included aromatase inhibitors and the estrogen receptor modulator, tamoxifen. Although some agents have been partially effective, the optimal pharmacologic treatment of PP in girls with MAS has not been identified. The objective of this study was to evaluate the safety and efficacy of fulvestrant (Faslodex^TM^), a pure estrogen receptor antagonist, in girls with progressive precocious puberty (PP) associated with McCune-Albright Syndrome (MAS).

**Methods:**

In this prospective international multicenter trial, thirty girls ≤ 10 years old with MAS and progressive PP received fulvestrant 4 mg/kg via monthly intramuscular injections for 12 months. Changes in vaginal bleeding, rates of bone age advancement, growth velocity, Tanner staging, predicted adult heights, and uterine and ovarian volumes were measured.

**Results:**

Median vaginal bleeding days decreased from 12.0 days per year to 1.0 day per year, with a median change in frequency of -3.6 days, (95% confidence interval (CI) -10.10, 0.00; p = 0.0146). Of patients with baseline bleeding, 74% experienced a ≥50% reduction in bleeding, and 35% experienced complete cessation during the study period (95% CI 51.6%, 89.8%; 16.4%, 57.3%, respectively). Average rates of bone age advancement (ΔBA/ΔCA) decreased from 1.99 pre-treatment to 1.06 on treatment (mean change -0.93, 95% CI -1.43, -0.43; p = 0.0007). No significant changes in uterine volumes or other endpoints or serious adverse events occurred.

**Conclusions:**

Fulvestrant was well tolerated and moderately effective in decreasing vaginal bleeding and rates of skeletal maturation in girls with MAS. Longer-term studies aimed at further defining potential benefits and risks of this novel therapeutic approach in girls with MAS are needed.

**Trial registration:**

NCT00278915

## Background

McCune-Albright syndrome (MAS) is characterized by the triad of peripheral precocious puberty (PP), fibrous dysplasia of bone, and café au lait spots [1,2]. This disorder develops secondary to a post-zygotic gain of function mutation in the gene encoding the alpha subunit of the heterotrimeric G-protein (Gsα) on chromosome 20, resulting in constitutive activation in affected cells [3,4].

PP, the most common manifestation of MAS, is diagnosed more frequently in girls than boys [5]. Autonomous activation of ovarian tissue leads to intermittent development of ovarian cysts, resulting in vaginal bleeding upon resolution and subsequent estrogen withdrawal [5,6]. A subset of girls develop progressive PP marked by recurrent vaginal bleeding, increased breast development, accelerated growth velocity, and bone age (BA) advancement with the potential for significant compromise in adult height [5]. Although the PP in MAS is gonadotropin independent, secondary activation of the hypothalamic-pituitary-gonadal axis may occur, resulting in concurrent central precocious puberty (CPP) [7].

Thus far, treatment options for PP in girls with MAS have met with mixed success. Fulvestrant (Faslodex^TM^) is a pure antiestrogen that binds to the estrogen receptor, triggering rapid degradation [8]. The objective of our study was to evaluate the safety and efficacy of fulvestrant in girls with progressive PP associated with MAS.

## Methods

This international prospective open-label trial recruited girls from 15 centers and was approved by an Institutional Review Board at each site. Because of the rarity of MAS, a study based on formal power calculations was not feasible. However, based on a similar AstraZeneca study evaluating tamoxifen in this population, an assumed proportion of 0.67 patients with a ≥ 50% reduction in vaginal episodes was used to determine that a group of 20 patients would generate a 95% confidence interval (CI) of 0.46-0.87 [9]. Thus, 30 patients were recruited with the goal of having 20 patients complete 12 months of treatment. Inclusion criteria included age ≤ 10 years at the start of therapy, and a diagnosis of MAS and progressive PP (onset before 8 years of age) made by a pediatric endocrinologist. MAS was diagnosed based on the presence of PP combined with café au lait spots, fibrous dysplasia, or a documented Gsα mutation. Subjects had clinical evidence of pubertal progression along with BA advancement (BA ≥ 12 months above chronologic age) or growth velocity > 2 standard deviations (SD) above the mean for age. Previously treated patients must have had documented progression on treatment with a 1 month washout period, or have stopped treatment for 6 months with subsequent progression of disease. Patients with CPP must have received at least 6 months of treatment with a gonadotropin-releasing hormone analog (GnRHa). Written informed consent of all parents/legal guardians and patient assent as locally required was obtained.

Patients were excluded if they had previously received fulvestrant, were currently receiving treatment for peripheral PP, had liver function tests ≥ 3 times the upper limit of normal, an International Normalized Ratio (INR) > 1.6, a history of bleeding diathesis or long-term anticoagulation, any severe comorbidities, or known hypersensitivity to any component of the study drug product.

Initial assessment occurred at a screening visit, followed by 13 monthly visits. Six months of pre-treatment data, including height, weight, Tanner stage, BA, and parental recall of vaginal bleeding history, were retrospectively reviewed. Physical exam including Tanner staging for breasts and pubic hair was performed at screening and at the 0, 3, 6, and 12 month visits.

BA radiographs were obtained at baseline, 6 and 12 months. Rate of skeletal maturation was defined as the change in BA divided by the change in chronological age. Pre-treatment BAs obtained at a minimum of 6 and maximum of 15 months apart were used to determine pre-treatment rates of skeletal maturation. Predicted adult heights (PAHs) were calculated based on the method of Bayley and Pinneau for patients 6 years or older. Vaginal bleeding data were obtained from patient diaries and reviewed monthly. Any missing days on diary cards were reported as bleeding days. Pelvic ultrasounds were obtained at the screening, 6 month, and 12 month visits. All radiographs were centrally read at Lifespan Health Research Center, at Wright State University, in Kettering, Ohio and all ultrasounds were read at Bio Clinica Inc., in Newton, Pennsylvania. Radiologists were blinded to patient diagnosis.

Chemiluminescent serum estradiol, testosterone, luteinizing hormone (LH), and follicle stimulating hormone (FSH) assays were obtained at screening and at 3, 6, and 12 months. Thyroxine and thyrotropin levels were drawn at baseline. Complete blood count, INR, alanine aminotransferase, and aspartate transaminase levels were obtained during screening. Liver function tests were repeated at the last visit. Laboratory assays were performed by Quintiles Laboratories North America (Marietta, GA).

The dose of fulvestrant was derived from studies in breast cancer patients, and was initiated at 2 mg/kg via monthly intramuscular injections in the first 10 patients [10]. Based on pharmacokinetics from the first 6 patients, the dose was increased to 4 mg/kg injections monthly, which corresponds to a dose of 250 mg/month in adult patients. All remaining patients received this dose for the entire study period. At 2 different time points between the 6 and 12 month visits, serum trough levels of medication were obtained to ensure that steady state drug concentrations were similar to those of patients in the breast cancer studies. Participants who completed the study were offered the option of continuing on treatment for an extension period with yearly data collection.

Intention-to-treat as well as per-protocol statistical analyses were performed. Results from both evaluations were consistent and so outcomes of the intention-to-treat analysis are reported. Analysis of hormone changes was performed retrospectively. For continuous efficacy endpoints with normal distributions, the mean changes from baseline to the treatment period were analyzed using the 2-sided paired t-test at the 5% level of significance, with a 95% CI using SAS PROC MEANS. A Signed rank test or a Sign test was used to analyze median changes (with SAS PROC UNIVARIATE) for endpoints with non-normal distributions. A distribution-free 95% CI of the median was obtained using SAS PROC UNIVARIATE with CIPCTLDF option. To evaluate the impact of outliers on BA advancement data, post-hoc analysis replacing the most extreme negative value with the second most extreme value was undertaken. No adjustments were made for multiple comparisons.

## Results

A total of 30 girls aged 5.86 ± 1.8 years were enrolled, of whom 29 completed 12 months of treatment. All patients had at least 2 of the classic components of MAS, and all had evidence of progressive PP. Three girls were receiving a GnRHa at baseline. Four girls had received aromatase inhibitors in the past, and none had been treated with tamoxifen. Twenty-three (77%) had vaginal bleeding during the pre-study interval. Baseline as well as pre-treatment participant characteristics are provided in Table 1.

**Table 1:** Baseline patient demographics (n = 30)

**Mean age ± SD (range)**	**5.86 years ± 1.8 (1.7-8.5)**
Ethnicity	
White	26 (87%)
Biracial	2 (7%)
Black	1 (3%)
Hispanic	1 (3%)
Polyostotic fibrous dysplasia (n)	21 (70.0%)
Café au lait spots (n)	24 (80.0%)
Confirmed Gsα mutation (n)	7 (23.3%)
Vaginal bleeding during 6 months pre-treatment (n)	23 (76.7%)
Median Tanner Stage for breasts (range)	III (I-IV)
Median Tanner Stage for pubic hair (range)	I (I-IV)
Mean growth velocity Z-score ± SD during 6 months prior to study	2.35 ± 3.3

SD-standard deviation.

Pharmacokinetic data revealed that patients on the 4 mg/kg dose reached steady-state serum concentrations consistent with patients effectively treated with fulvestrant for breast cancer. The mean serum half-life of the drug was 70.4 ± 8.1 days.

**Figure 1:**
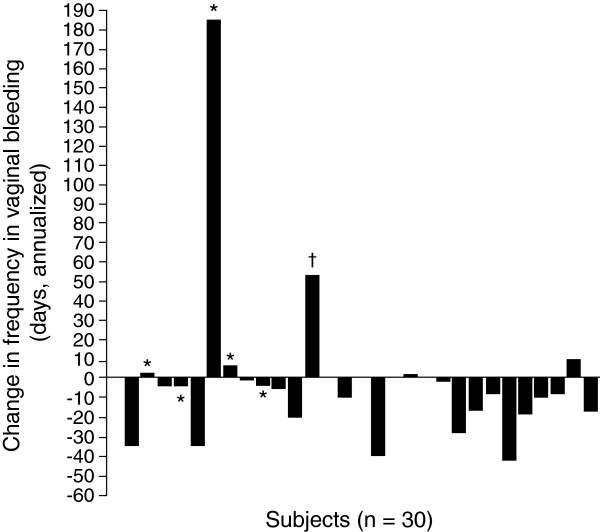
**Change in frequency of annualized days of vaginal bleeding from pre-treatment to on- treatment period.** Bleeding was calculated based on worst-case scenario (assuming bleeding occurred on days without diary data available). *Patients with one or more missing days of data which were counted as bleeding days. ^†^Patients withdrew from study due to worsening of condition.

Median vaginal bleeding days on treatment decreased from 12.0 days per year to 1.0 day per year, with a median change in frequency of -3.6 days (p = 0.0146). Of the patients with baseline bleeding, 17 (74%) experienced a ≥50% reduction in bleeding, and 8 (35%) experienced complete cessation during the year of study (CI 51.6%, 89.8%; 16.4%, 57.3%, respectively). One patient was withdrawn due to worsening of her condition after receiving 6 injections. Figure 1 depicts individual changes in vaginal bleeding.

**Figure 2:**
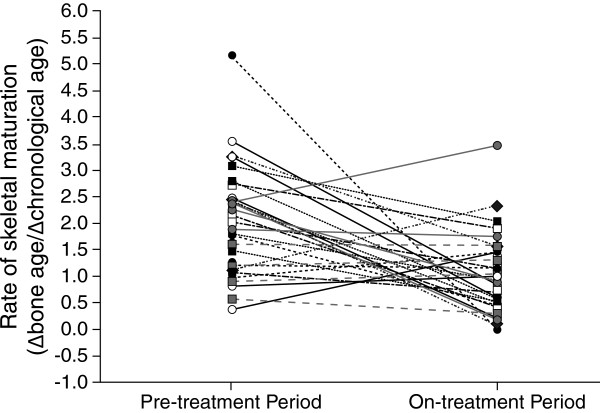
**Change in skeletal maturation.** Rate of skeletal maturation during pre-treatment (change in BA during 6 months prior to treatment) and on-treatment period (change in BA during the 12 months of study) by patient (n = 30).

Average rates of BA advancement decreased from 1.99 at baseline to 1.06 after 12 months of treatment, a difference of 0.93 ± 1.3 (95% CI -1.43, -0.43; p = 0.0007), when compared with the pre-treatment interval. Furthermore, this was progressive, as the mean BA advancement over the first 6 months of treatment decreased by 0.83 compared to pretreatment (95% CI -1.39, -0.26; p =0.005), while over the second 6 months, a more substantial decrease of 1.10 was seen (95% CI -1.63, -0.58; p = 0.0002). Individual changes in skeletal maturation are illustrated in Figure 2. No statistically significant difference was seen in mean growth velocity z-scores during treatment compared with the pre-treatment interval (mean change -1.14, 95% CI -2.67, 0.38; p = 0.135). Mean PAHs before and after treatment were equivalent (163.0 ± 6.9 cm vs. 163.5 ± 6.3 cm).

Hormone levels are presented in Table 2. Patients experienced a significant decrease in LH on treatment, with a median difference of 0.35 IU/L from baseline to 12 months (CI 0, 0.8; p < 0.005). No girls developed CPP during the study period. Mean uterine volume at baseline was 8.2 ± 5.0 ml, corresponding to a pubertal size. No significant difference was seen in uterine size during the 12 months of study. No significant changes in other hormones, Tanner staging, ovarian cysts, or ovarian volumes were seen throughout the study period.

**Table 2:** Mean hormone levels

**Hormone (units)**	**Visit**	**n**	**Mean ± SD**	**Median**
Free thyroxine (pmol/L)	Screening	30	14.42 ± 2.53	14.65
Thyrotropin (mIU/L)	Screening	30	2.38 ± 3.30	1.53
Estradiol (pmol/L)	Screening	30	20.54 ± 25.59	9.18
	6 month visit	29	36.10 ± 104.95	9.18
	12 month visit	26	25.95 ± 30.72	9.55
LH (IU/L)*	Screening	30	0.84 ± 1.04	0.45
	6 month visit	29	0.10 ± 0.02	0.10
	12 month visit	28	0.11 ± 0.04	0.10
FSH (IU/L)	Screening	30	3.86 ± 5.42	1.95
	6 month visit	29	0.82 ± 0.78	0.50
	12 month visit	29	1.13 ± 1.02	0.60
Testosterone (nmol/L)	Screening	30	0.53 ± 0.19	0.48
	6 month visit	29	0.56 ± 0.24	0.45
	12 month visit	28	0.65 ± 0.27	0.66

Prepubertal normal ranges: free thyroxine 10.3-25.7 pmol/L; thyrotropin 0.7-6.4 mIU/L; estradiol < 73.4 pmol/L; LH 0.6-1.6 IU/L; FSH 0.7-6.7 IU/L; testosterone < 0.35-1.2 nmol/L.

* P < 0.005 for median change from baseline to month 12.

Fulvestrant was generally well tolerated. Seven patients reported injection site reactions that were typically short lived, but recurred in over half of cases. These included mild hematoma and rash as well as mild-moderate inflammation. Vomiting and abdominal pain possibly related to fulvestrant were each reported in 1 patient. No serious treatment-related adverse events occurred and no patients discontinued the study secondary to adverse events. Of the 29 patients completing the study, 24 girls chose to enter the extension phase and continue treatment.

## Discussion

The ideal treatment of PP in girls with MAS remains elusive. Medroxyprogesterone and cyproterone acetate can be effective for alleviation of vaginal bleeding, but have no effect on BA advancement [11,12]. Ketoconazole has been reported to result in cessation of bleeding and regression of secondary sexual characteristics in case studies, but lack of long-term data and concerns for risk of adrenal insufficiency and hepatotoxicity have limited its use. Thus, recent interest has revolved around the use of antiestrogens [13-15].

Aromatase inhibitors (AIs) function by binding to the cytochrome P450 portion of aromatase, inhibiting the conversion of androgens to estrogens [16]. While this class of drugs initially showed promise in MAS, long-term studies of the first generation AI testolactone revealed no significant improvement in skeletal maturation [16-18]. Later generation agents with increased potency have similarly failed to indicate significant benefit in prospective trials, with the exception of letrozole [19-21]. While this third generation AI did have a positive effect on indices of PP in a small study, an increase in mean ovarian volumes and occurrence of ovarian torsion in one patient have raised concerns regarding the safety of this drug [21].

Tamoxifen, a selective estrogen receptor modulator widely used in breast cancer therapy, binds to the estrogen receptor and only partially triggers the normal activating sequence, thereby attenuating transcription [8]. While a prospective study of this medication in girls with MAS and PP demonstrated decreases in vaginal bleeding, growth velocities, and skeletal maturation, a progressive increase in uterine volume was observed during the treatment period [9]. Although the significance of this finding remains unknown, it is of concern given previous links to endometrial stromal tumor development in women undergoing tamoxifen therapy [22].

Fulvestrant was also developed as a treatment for breast cancer subsequent to its effects at the level of the estrogen receptor. Because of its purely antagonistic properties, the partial estrogen agonistic actions seen with tamoxifen should hypothetically be avoided [8]. This was supported by lack in changes in uterine or ovarian dimensions on treatment. To our knowledge, no previous reports utilizing fulvestrant in pediatric patients exist. This study demonstrated that fulvestrant significantly decreased vaginal bleeding and reduced rates of skeletal maturation to near normal in this population. However, complete cessation of vaginal bleeding occurred in only a third of subjects, and no significant change in growth velocity or PAH was seen. A progressive decrease in BA advancement with drug exposure supports that a longer treatment interval might improve these parameters. The etiology for the observed decrease in LH levels is unclear, but may have been temporally related to episodic autonomous ovarian activation, which would not be expected to be altered by fulvestrant.

There are several limitations to our study. Because of the rarity of MAS, each girl served as her own control. However, the inclusion of multiple centers enabled a reasonable sample size and generated statistically significant results. A conservative, worst-case scenario approach was applied to missing data for vaginal bleeding diaries that could potentially underestimate the benefits of treatment. Pre-treatment vaginal bleeding data was collected retrospectively and therefore may not have been as accurate as data collected during the treatment period. Conversely, concurrent MAS-related endocrinopathies and skeletal deformities resulting from fibrous dysplasia could have affected growth velocities, rates of skeletal maturation, and accuracy of height measurements. The lack of change in growth velocities and PAH could reflect an insufficient treatment interval or other aspects of MAS unrelated to PP.

## Conclusion

In conclusion, fulvestrant was moderately effective in decreasing vaginal bleeding and rates of skeletal maturation in girls with progressive PP secondary to MAS over a 1 year period. The medication was well tolerated. Longer follow-up of patients receiving treatment will be necessary in order to confirm these results.

## Abbreviations

PP: Precocious puberty; MAS: McCune-Albright Syndrome; CI: Confidence interval; Gsα: Alpha subunit of the heterotrimeric G-protein; BA: Bone age; CPP: Central precocious puberty; SD: Standard deviation; GnRHa: Gonadotropin-releasing hormone analog; INR: International normalized ratio; PAH: Predicted adult height; LH: Luteinizing hormone; FSH: Follicle stimulating hormone; AI: Aromatase inhibitor.

## Competing interests

This study was supported by AstraZeneca and involves off-label use of fulvestrant for the treatment of precocious puberty in girls with McCune-Albright syndrome. The role of the sponsor involved an AstraZeneca statistician participating in analysis of the study data and approval of the final manuscript. EKS, FP and CS have no conflicts of interest to declare. EAE has served as a consultant for AstraZeneca, and reports this as a potential conflict of interest. SG is currently employed by AstraZeneca and FG is a former employee, and these authors report these roles as potential conflicts of interest.

## Authors’ contributions

EKS participated in analysis and interpretation of the data, wrote the first draft of the manuscript, revised the manuscript for intellectual content, and approved the final manuscript as submitted. SG analyzed the data, participated in the revision of the manuscript, and approved the final manuscript as submitted. FG participated in the design of the study and coordination of study results, analyzed the data, revised the manuscript for intellectual content, and approved the final manuscript as submitted. FP participated in interpretation of the data, revised the manuscript for intellectual content, and approved the final manuscript as submitted. CS participated in interpretation of the data, revised the manuscript for intellectual content, and approved the final manuscript as submitted. EAE participated in interpretation of the data, revised the manuscript for intellectual content, and approved the final manuscript as submitted. All authors read and approved the final manuscript.
